# Body shape phenotype, TYG trajectory and the risk of digestive system cancers

**DOI:** 10.3389/fnut.2025.1658356

**Published:** 2025-09-23

**Authors:** Zixiang Kai, Chenan Liu, Qingsong Zhang, Shouling Wu, Keming Yun

**Affiliations:** ^1^School of Forensic Medicine, Shanxi Medical University, Jinzhong, China; ^2^Shanxi Key Laboratory of Forensic Medicine, Jinzhong, China; ^3^Key Laboratory of Forensic Toxicology of Ministry of Public Security, Jinzhong, China; ^4^Department of Comprehensive Oncology, National Cancer Center/National Clinical Research Center for Cancer/Cancer Hospital, Chinese Academy of Medical Sciences and Peking Union Medical College, Beijing, China; ^5^Department of General Surgery, Kailuan General Hospital, Tangshan, China; ^6^Department of Cardiology, Kailuan General Hospital, Tangshan, China

**Keywords:** triglyceride-glucose index, body shape, principal component analysis, colorectal cancer, gastric cancer

## Abstract

**Background:**

The triglyceride-glucose (TyG) index and unhealthy body shape have been shown to indicate the level of insulin resistance in the body and are associated with various chronic diseases. However, the longitudinal pattern of change in relation to the risk of digestive system cancers remains unclear.

**Methods:**

All participants were drawn from a large prospective cohort study, the Kailuan Study. Latent mixture modeling was used to identify similar shared TyG index trajectories among participants who underwent annual physical examinations between 2006 and 2010. Principal component analysis was used to evaluate the body shape characteristics of participants. Cox regression models were used to investigate the relationship between the TyG index trajectories and the risk of digestive system cancers.

**Results:**

A total of 53,350 participants were included in the study, with an average age of 49.5 (11.7) years. Five distinct TyG index trajectories were identified. During a median follow-up of 11.0 years, 804 participants developed digestive system cancer. Four characteristic phenotypes of body type were identified. Compared with a single measurement of the TyG index, TyG index trajectories better predicted the risk of digestive system cancer. After adjusting for potential confounders, the hazard ratios for developing digestive system cancers in the moderate low-stable, moderate high-stable, high-stable, and high-increasing groups compared with those of the low-stable group were 1.16, 1.42, 1.45, and 2.48, respectively. Consistently, as the trajectory changes, the components loads of body shape also constantly changes. Similar trends were observed for the risk of colorectal and gastric cancer.

**Conclusion:**

The TyG index trajectory is better than a single TyG index measurement for predicting the risk of digestive system cancers. Long-term maintenance of a high TyG index trajectory is associated with a less healthy body shape phenotype and an increased risk of digestive system cancers, especially colorectal cancer and gastric cancer.

## Introduction

Digestive system cancers are a major global health threat, causing a significant disease burden in both developing and developed countries (1, 2). Various digestive system cancers, such as colorectal, gastric, pancreatic, and liver cancers, exhibit different epidemiological trends in different regions; however, they share a common challenge, which is the difficulty of early diagnosis and the effectiveness of treatment (3). Therefore, the search for new biomarkers or predictive factors to accurately identify cancer risk is of paramount importance for prevention and early intervention.

In recent years, an increasing number of studies have linked metabolic factors to cancer risk. The triglyceride-glucose (TyG) index, a composite indicator of triglycerides (TG) and fasting plasma glucose (FBG), reflects the body’s insulin resistance status and has been linked to metabolic diseases (e.g., obesity, type 2 diabetes) and digestive system cancer risk in cross-sectional or single-measurement prospective studies (4–7). Fritz et al. (8) also reported that the TyG index can serve as a good indicator of insulin resistance and is closely associated with the risk of digestive system- and obesity-related cancers. However, most existing studies rely on one-time TyG measurements, failing to capture its dynamic changes over time—an important limitation, as TyG is highly susceptible to temporal factors (e.g., diet, medication) that may affect its association with long-term cancer risk (9). This knowledge gap highlights the need to explore the longitudinal trajectory of TyG and its relationship with digestive system cancer incidence.

In addition, body anthropometry-defined body types have been proven to be associated with digestive system cancers. Lee et al. demonstrated that abdominal obesity, as indicated by an increase in waist circumference (WC), is linked to an increased risk of 18 types of cancers, including colorectal and gastric cancers (10). Abnormal insulin metabolism is considered one of the significant reasons for unhealthy body shapes (11). However, the dynamic changes in insulin resistance and how they influence body shape phenotype, leading to digestive system cancers, remain unknown to us.

This study aimed to investigate the potential association between the TyG index, body shape phenotypes, and the incidence of digestive system cancers in a large-scale prospective cohort. The goal is to provide new insights into the early prediction, intervention, and personalized treatment of cancer.

## Methods

### Study design and participants

The study population was drawn from the Kailuan Study, a community-based prospective cohort study initiated in July 2006. Between 2006 and 2007, 11 hospitals affiliated with Kailuan General Hospital conducted clinical baseline assessments, health questionnaires, laboratory tests, and imaging examinations of employees of the Kailuan Group aged 18 to 98 years (12). At the time of the survey, each participant received a detailed introduction to the project and provided written informed consent. A total of 101,510 participants were included in this study. Subsequent follow-up measurements were conducted every 2 years for these participants.

In this study, because of the need to construct TyG trajectories based on clinical data from 2006 to 2010, we initially included 57,927 participants who underwent three physical examinations during this period (in 2006, 2008, and 2010). Subsequently, we excluded the following: (1) Individuals lacking TG and FBG required for TyG index calculation; (2) Individuals with a history of or current cancer; (3) Individuals lacking information on covariates, such as age, sex, marital status, educational level, type of work, regular physical activity, sedentary behavior, smoking habits, alcohol use, body mass index (BMI), waist circumference, hypertension, diabetes mellitus, family history of cancer, C-reactive protein, salt consumption, total cholesterol (TC), triglycerides (TG), gallstone disease, cirrhosis, hepatitis B virus (HBV) infection, and other relevant covariates; and (4) Individuals with multiple primary site cancers.

**Figure 1:**
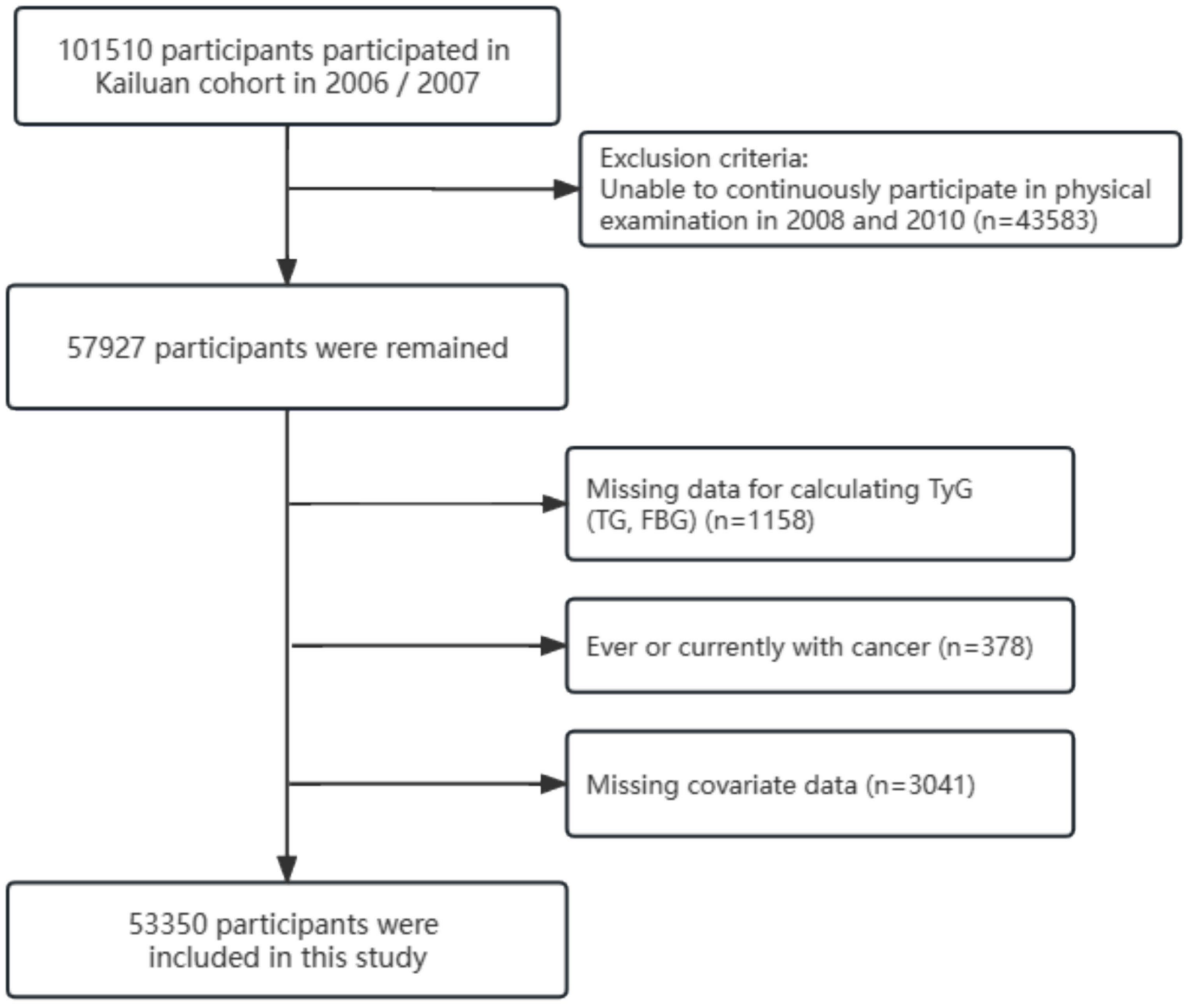
The flowchart of this study.

Ultimately, 53,350 participants were included in this study. The detailed screening process is illustrated in Figure 1. Both the cohort study and present analysis adhered to the Helsinki Declaration and were approved by the Ethics Committees of Kailuan General Hospital.

### Assessment of the TyG index and covariates

The TyG index was calculated based on a previously validated formula: TyG = ln (TG [mg/dL] × FBG [mg/dL] / 2) (13). The covariates were reviewed. Marital status was categorized as married or single (divorced, never married, or widowed). Educational level was divided into college and above, and below college level. Occupation was categorized as manual labor or mental work. Sedentary behavior was classified as sedentary or non-sedentary based on an 8-h threshold. Regular physical exercise was considered as physical exercise ≥ 3 times/week for ≥30 min/time. After fasting for at least 8 h, the participants visited the hospital in the morning for venous blood collection. These blood samples were then sent to the central laboratory at Kailuan General Hospital, where a Hitachi 747 fully automated analyzer was used to measure C-reactive protein (CRP), TC, TG, and FBG (14). Additionally, quantitative detection of Hepatitis B surface antigen (HBsAg) was performed using an enzyme-linked immunosorbent assay kit from Shanghai Kehua Bio-engineering (KHB, Shanghai, China). Liver cirrhosis and gallstones were diagnosed using real-time abdominal ultrasound examinations of each participant by two ultrasound physicians using a Philips HD-15 ultrasound machine (15). Diabetes was defined as having an FBG ≥ 7.0 mmol/L, a history of oral hypoglycemic agent or insulin use, or a previous diagnosis of diabetes. Hypertension was defined as having a systolic blood pressure of ≥140 mmHg and/or a diastolic blood pressure of ≥90 mmHg, using antihypertensive medications, or having a previous diagnosis of hypertension. All covariates were recorded in 2010.

### Assessment of the body anthropometric measures

Just as described in previous studies (16), we requested all patients to undergo measurements of waist circumference (WC), hip circumference (HC), height (HT), weight (WT), body mass index (BMI), and waist-hip ratio (WHR) after removing outerwear and shoes. During the measurement process, we conducted three repeated measurements for each participant, and the average was recorded. BMI is calculated as WT (kg) divided by the square of HT (m), while WHR is the ratio of WC to HC.

### Assessment of digestive system cancers

The occurrence of digestive system cancers was the primary outcome and was assessed using several methods, including: (1) Clinical records with radiological or pathological evaluations; (2) Questionnaires, which included inquiries about whether the participant had been diagnosed with cancer, the date of diagnosis, and the cancer site; (3) Tangshan City medical insurance system or the Kailuan Social Security Information System records, which covered all participants. According to the International Classification of Diseases, Tenth Revision (ICD-10), cases of digestive system cancer were categorized as follows: esophageal cancer (C15), gastric cancer (C16), small intestine cancer (C17), colorectal cancer (C18–21), liver cancer (C22), gallbladder and extrahepatic bile duct cancer (C23–24), and pancreatic cancer (C25). The last follow-up date was December 31, 2021, or the last contact date with the participant.

### Statistical analysis

The statistical analysis was conducted using R version 4.2.0 (R Foundation for Statistical Computing, Vienna, Austria) and SAS version 9.4 (SAS Institute, Cary, NC, United States). Two-tailed *p*-values less than 0.05 were considered statistically significant.

The TyG trajectories were constructed based on the values obtained from all participants in 2006, 2008, and 2010. The “PROC TRAJ” function in SAS software and latent mixture modeling were used to identify potential similar TyG change trajectories. The model was constructed in a stepwise manner. Initially, we established models for five trajectories in a quadratic form and compared them with models with 4, 3, 2, and 1 trajectories. Subsequently, we compared the significance levels of the intercept, linear, and quadratic terms (17). The best trajectory model was selected using the Bayesian Information Criterion (Supplementary Table S1). In this study, we determined that the model with 5 trajectories was the optimal TyG index trajectory model.

According to the recommendations from previous study, we utilized WC, WT, HT, BMI, HC, and WHR for principal component analysis (PCA) of body shape phenotypes (18). PCA is a commonly used method for data dimensionality reduction, effectively distinguishing complex body measurement indicators into distinct phenotypes. We constructed six principal components (PC), each variable having different weights in each PC. Based on the cumulative explained variance, we selected the top 4 PC for further analysis (Supplementary Table S2). To better understand the meaning of each component, we presented some characteristic populations using the^1^ website (Supplementary Figure S1).

Continuous variables that followed a normal distribution were reported as the mean ± standard deviation (SD), otherwise as the median and interquartile range (IQR). Categorical variables were reported as frequencies and percentages. Intergroup differences were compared using one-way analysis of variance (ANOVA), the Kruskal–Wallis test, or the chi-squared test, depending on the data type. To assess the predictive ability of the TyG index trajectories, TyG in 2006, TyG in 2008, and TyG in 2010 for digestive tract cancer, we used the integrated discrimination improvement (IDI) and net reclassification improvement (NRI) metrics. Cox proportional hazards models were used to explore the associations between different trajectories and digestive system cancer risk, with results reported as hazard ratios (HR) and 95% confidence intervals (CI). Subgroup and interaction analyses were performed to explore potential confounding factors in the association between TyG and the risk of digestive system cancer. Restricted cubic spline (RCS) curves were used to explore the linear association between the TyG index and digestive system cancer risk. Sensitivity analyses were conducted to validate the robustness of the results, including: (1) Excluding participants with cancer diagnosed within the first year of follow-up to avoid causality; (2) Excluding participants taking antihypertensive, lipid-lowering, or antidiabetic medications to avoid fluctuations in the TyG index due to drug treatment; (3) Additional adjustment for the TyG index in 2010; (4) Excluding participants with HBV infection, as HBV has been closely associated with digestive system cancer (15); (5) Competing risk models, including cause-specific risk models and subdistribution hazard models, to account for the potential bias caused by death as a competing event (19).

## Results

### Baseline characteristics

**Figure 2:**
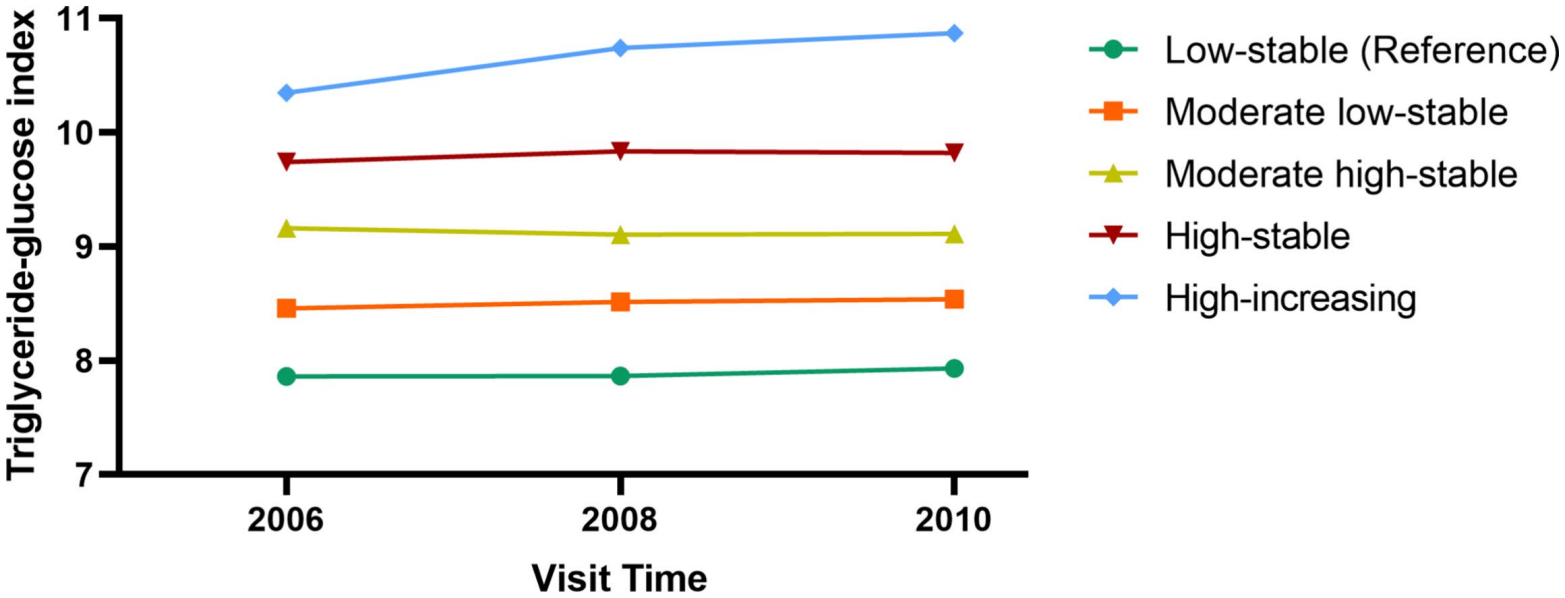
Triglyceride-glucose index trajectory during 2006–2010.

A total of 53,350 participants were included in this study, with a mean age of 49.5 (11.7) years, of whom 40,945 were male and 12,405 were female. Five different TyG trajectories were identified as follows (Figure 2): low-stable group (*n* = 9,252; range 7.86–7.93), moderate low-stable group (*n* = 25,905; range 8.45–8.54), moderate high-stable group (*n* = 13,832; range 9.10–9.16), high-stable group (*n* = 3,752; range 9.74–9.83), and high-increasing group (*n* = 609; range 10.34–10.77). Compared with the low-stable group, the high-increasing group were younger, more likely to be male, with a higher BMI, larger 2, lower educational level, more sedentary behavior, less regular physical activity, higher rates of smoking and alcohol consumption, higher prevalence of hypertension and diabetes, and elevated CRP, TC, and TG levels (Table 1).

**Table 1:** Baseline characteristics of participants according to the trajectories of TyG index.

Characteristics	level	Low-stable	Moderate low-stable	Moderate high-stable	High-stable	High-increasing	p
*N*		9,252	25,905	13,832	3,752	609	
Age (Year)		49.99 (12.73)	49.36 (12.01)	49.63 (11.13)	48.83 (10.28)	47.42 (9.14)	<0.001
Sex (%)	Men	6,216 (67.2)	19,764 (76.3)	11,281 (81.6)	3,151 (84.0)	533 (87.5)	<0.001
BMI (kg/m^2^)		23.15 (3.15)	24.66 (3.29)	25.98 (3.29)	26.46 (3.25)	26.59 (3.40)	<0.001
BMI (%)	<18.5 kg/m^2^	5,972 (64.5)	10,891 (42.0)	3,250 (23.5)	666 (17.8)	95 (15.6)	<0.001
	10.5–23.9 kg/m^2^	2,642 (28.6)	10,868 (42.0)	6,755 (48.8)	1808 (48.2)	296 (48.6)	
	≥24 kg/m^2^	638 (6.9)	4,146 (16.0)	3,827 (27.7)	1,278 (34.1)	218 (35.8)	
Waist circumference (cm)		83.0 [76.0,90.0]	87.0 [81.0,94.0]	90.3 [85.0,97.0]	92.0 [87.0,98.0]	93.0 [87.0,98.0]	<0.001
Marital status	Married	9,029 (97.6)	25,401 (98.1)	13,617 (98.4)	3,679 (98.1)	601 (98.7)	<0.001
Education (%)	College or above	2,678 (28.9)	7,013 (27.1)	3,586 (25.9)	922 (24.6)	141 (23.2)	<0.001
Type of work (%)	Physical labor	7,443 (82.6)	21,086 (85.1)	11,290 (85.4)	3,133 (86.8)	500 (86.2)	<0.001
Sedentary time (%)	≥8 h	2,509 (27.1)	6,520 (25.2)	3,576 (25.9)	1,042 (27.8)	173 (28.4)	<0.001
Regular physical activity (%)	Yes	1,384 (15.0)	3,888 (15.0)	2023 (14.6)	482 (12.8)	65 (10.7)	<0.001
Smoke (%)	Yes	2,406 (26.0)	7,647 (29.5)	4,743 (34.3)	1,414 (37.7)	266 (43.7)	<0.001
Alcohol use (%)	Yes	1,364 (14.7)	4,380 (16.9)	2,836 (20.5)	913 (24.3)	159 (26.1)	<0.001
Hypertension (%)	Yes	2,230 (24.1)	9,805 (37.8)	6,712 (48.5)	1988 (53.0)	332 (54.5)	<0.001
Diabetes mellitus (%)	Yes	75 (0.8)	895 (3.5)	1953 (14.1)	1,082 (28.8)	296 (48.6)	<0.001
Family history of tumor (%)	Yes	387 (4.2)	1,087 (4.2)	611 (4.4)	163 (4.3)	19 (3.1)	0.520
CRP (mg/L)		0.90 [0.47,2.07]	1.00 [0.47,2.25]	1.20 [0.56,2.76]	1.49 [0.70,3.27]	1.69 [0.89,3.50]	<0.001
CRP (%)	≥2 mg/L	1,599 (17.3)	4,875 (18.8)	3,195 (23.1)	1,042 (27.8)	182 (29.9)	<0.001
Salt consumption (%)	≥10 g/d	777 (8.4)	2,633 (10.2)	1,559 (11.3)	461 (12.3)	92 (15.1)	<0.001
Total cholesterol (mmol/L)		4.51 [4.00,5.08]	4.87 [4.30,5.47]	5.09 [4.54,5.77]	5.32 [4.71,6.05]	6.13 [5.25,7.06]	<0.001
Triglyceride (mmol/L)		0.70 [0.56,0.87]	1.20 [0.96,1.50]	1.92 [1.42,2.57]	3.38 [2.34,4.91]	7.13 [4.56,11.65]	<0.001
Gallstone disease (%)	Yes	204 (2.2)	564 (2.2)	337 (2.4)	72 (1.9)	14 (2.3)	0.316
Cirrhosis (%)	Yes	77 (0.8)	203 (0.8)	100 (0.7)	25 (0.7)	6 (1.0)	0.767
Hepatitis B virus infection (%)	Yes	370 (4.0)	673 (2.6)	304 (2.2)	68 (1.8)	8 (1.3)	<0.001

### Associations between TyG index trajectories and the body shape phenotypes

**Figure 3:**
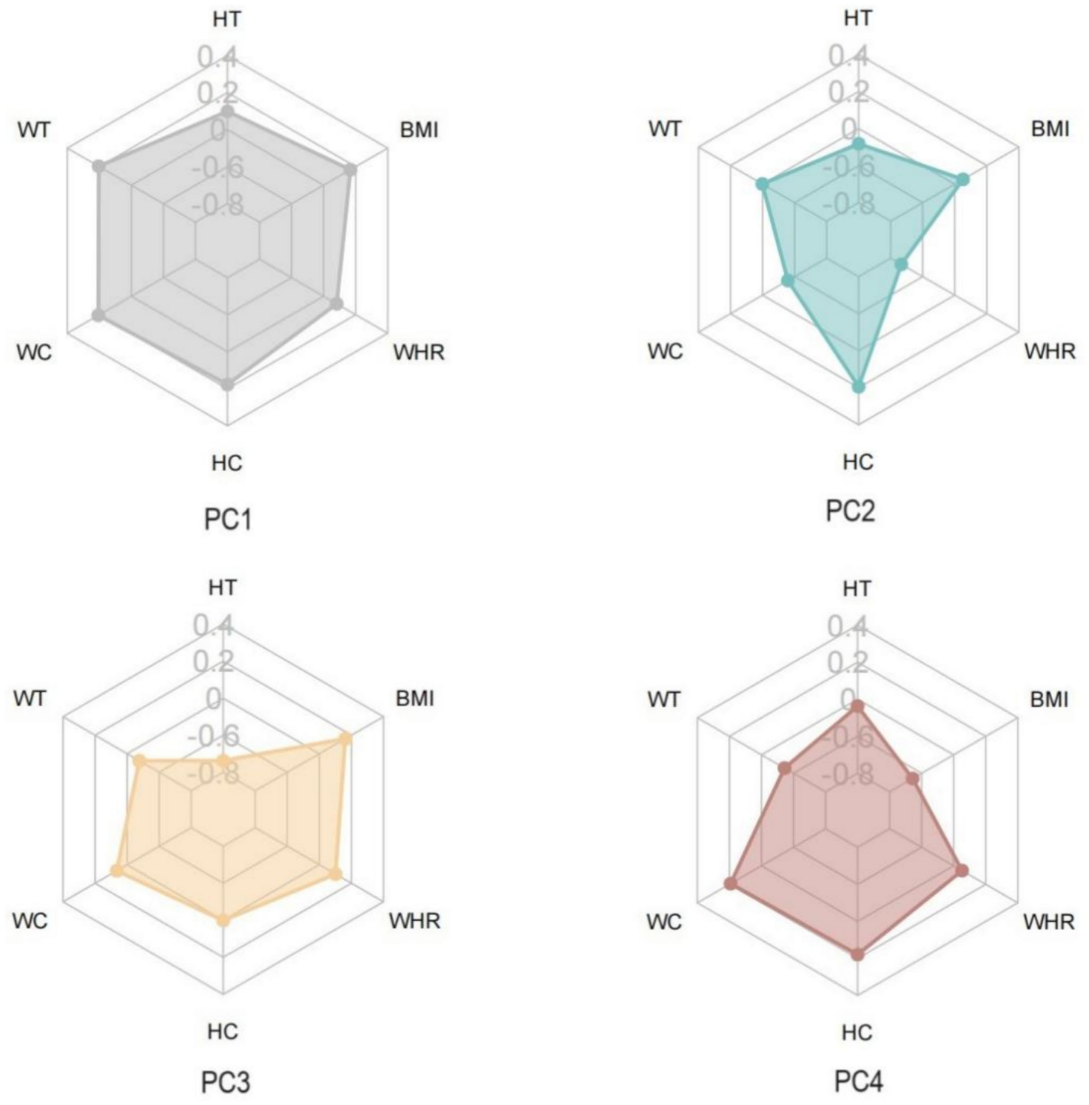
Components loads of phenotypes of body shape.

Although there are six identified principal components (PC), the top four PC collectively explain approximately 99.10% of the variability in variables. Therefore, we selected these four distinct PCs for analysis. These four PCs are PC1 (tall stature), PC2 (Low WC and WHR, High HC), PC3 (Low HT, High BMI, WC, and WHR), and PC4 (High WC, HC, and WHR, Low BMI) (Figure 3). To further explore the associations between TyG trajectories and PCs, we assigned scores for each PC to every participant. Compared to participants in the low-stable group, patients in the remaining four TyG trajectory groups showed gradual increases in PC1 and PC3 scores, along with a gradual decrease in PC2 scores (Supplementary Figure S2).

### Associations between TyG index trajectories and the risk of digestive system cancers

We initially explored the association between a single TyG index measurement and the risk of digestive system cancers. The RCS results indicated that as TyG levels increased, the risk of digestive system cancer increased linearly (Supplementary Figure S3). Subsequently, we compared the performance of different forms of the TyG index for predicting the risk of digestive system cancers using the IDI and NRI, as shown in Supplementary Table S3. Compared with TyG index trajectories, TyG in 2006 (IDI: −0.006 [−0.005,-0.007], NRI:-0.001 [−0.001,0]), 2008 (IDI:-0.003 [−0.005,-0.002], NRI: −0.001 [−0.001,0]), and 2010 (IDI: −0.001 [−0.001,0], NRI: −0.002 [−0.003,0])had were less able to predict cancer risk.

After adjusting for potential confounding factors (Table 2), participants in the moderate high-stable group (HR: 1.42, 95% CI: 1.12–1.81), high-stable group (HR: 1.45, 95% CI: 1.05–2.01), and high-increasing group (HR: 2.48, 95% CI: 1.45–4.22) had an increased risk of developing digestive system cancers than did those in the low-stable group. Regarding specific cancer types, TyG index trajectories were significantly associated with colorectal and gastric cancer risk. Compared with the low-stable group, the risk of colorectal cancer was significantly increased in the moderate low-stable (HR: 1.57, 95% CI: 1.03–2.41), moderate high-stable (HR: 1.77, 95% CI: 1.11–2.81), high-stable (HR: 2.21, 95% CI: 1.12–3.69), and high-increasing (HR: 3.11, 95% CI: 1.15–8.40) trajectory groups. Compared with the low-stable group, the risk of gastric cancer was significantly increased in the moderate high-stable (HR: 1.70, 95% CI: 1.03–2.87), high-stable (HR: 2.19, 95% CI: 1.13–4.25), and high-increasing (HR: 3.15, 95% CI: 1.03–9.62) trajectory groups.

**Table 2:** The hazard ratio (HR) of digestive system cancer according to trajectories of TyG index.

Trajectories of TyG index	Case	Crude HR (95%CI)	p	Adjusted HR (95%CI)^*^	p
Digestive system cancers					
Low-stable	106	Ref.		Ref.	
Moderate low-stable	372	1.171 (0.944,1.453)	0.152	1.158 (0.931,1.442)	0.186
Moderate high-stable	244	1.455 (1.158,1.827)	0.001	1.423 (1.120,1.809)	0.003
High-stable	65	1.497 (1.099,2.041)	0.011	1.451 (1.046,2.014)	0.025
High-increasing	17	2.574 (1.541,4.301)	<0.001	2.477 (1.452,4.224)	<0.001
Colorectal cancer					
Low-stable	26	Ref.		Ref.	
Moderate low-stable	124	1.597 (1.046,2.437)	0.030	1.571 (1.025,2.409)	0.038
Moderate high-stable	74	1.810 (1.157,2.830)	0.009	1.767 (1.109,2.810)	0.016
High-stable	22	2.084 (1.180,3.680)	0.011	2.208 (1.115,3.691)	0.021
High-increasing	5	3.156 (1.210,8.235)	0.019	3.114 (1.154,8.404)	0.025
Gastric cancer					
Low-stable	21	Ref.		Ref.	
Moderate low-stable	79	1.262 (0.780,2.042)	0.343	1.225 (0.753,1.934)	0.413
Moderate high-stable	58	1.786 (1.083,2.943)	0.023	1.701 (1.029,2.870)	0.046
High-stable	19	2.305 (1.237,4.296)	0.009	2.192 (1.131,4.249)	0.002
High-increasing	4	3.273 (1.121,9.561)	0.030	3.151 (1.033,9.615)	0.043
Hepatocellular carcinoma					
Low-stable	33	Ref.		Ref.	
Moderate low-stable	77	0.772 (0.513,1.161)	0.214	0.707 (0.465,1.074)	0.104
Moderate high-stable	58	1.082 (0.705,1.660)	0.719	0.942 (0.593,1.496)	0.800
High-stable	8	0.565 (0.261,1.225)	0.148	0.438 (0.194,1.077)	0.147
High-increasing	3	1.349 (0.413,4.409)	0.619	0.911 (0.265,3.127)	0.882
Esophageal cancer					
Low-stable	11	Ref.		Ref.	
Moderate low-stable	45	1.331 (0.689,2.575)	0.395	1.266 (0.664,2.413)	0.474
Moderate high-stable	26	1.418 (0.700,2.875)	0.332	1.674 (0.804,3.487)	0.168
High-stable	9	1.846 (0.762,4.470)	0.174	2.255 (0.895,5.686)	0.084
High-increasing	2	2.580 (0.570,11.681)	0.219	3.301 (0.696,15.651)	0.133
Pancreatic cancer					
Low-stable	8	Ref.		Ref.	
Moderate low-stable	28	1.178 (0.537,2.585)	0.682	1.126 (0.506,2.504)	0.771
Moderate high-stable	15	1.218 (0.516,2.876)	0.652	1.271 (0.517,3.126)	0.601
High-stable	4	1.288 (0.387,4.289)	0.679	1.451 (0.408,5.159)	0.565
High-increasing	1	2.196 (0.273,17.636)	0.459	2.744 (0.317,23.741)	0.359
Gallbladder or extrahepatic bile duct cancer					
Low-stable	2	Ref.		Ref.	
Moderate low-stable	11	1.848 (0.410,8.337)	0.424	1.879 (0.411,8.596)	0.416
Moderate high-stable	5	1.634 (0.316,8.430)	0.558	1.829 (0.338,9.907)	0.483
High-stable	1	1.310 (0.118,14.501)	0.826	1.781 (0.152,20.967)	0.646
High-increasing	2	8.176 (2.526,13.749)	0.004	9.782 (3.976,20.798)	0.001
Small intestine cancer					
Low-stable	5	Ref.		Ref.	
Moderate low-stable	8	0.532 (0.174,1.628)	0.269	0.469 (0.150,1.466)	0.193
Moderate high-stable	8	1.007 (0.328,3.093)	0.989	0.764 (0.228,2.588)	0.666
High-stable	2	0.971 (0.187,5.046)	0.972	0.923 (0.162,5.250)	0.928
High-increasing	0	N/A		N/A	

^*^Adjusted model was adjusted for age, sex, marital status, educational level, type of work, regular physical activity, sedentary, smoke, alcohol use, BMI, waist circumference, hypertension, diabetes mellitus, family history of cancer, CRP, salt consumption, TC, TG, gallstone disease, cirrhosis, HBV infection.

Subsequently, we conducted subgroup analyses of digestive system (Supplementary Table S4), colorectal (Supplementary Table S5), and gastric (Supplementary Table S6) cancers. After stratification by potential influencing factors, TyG index trajectories were closely associated with the risk of digestive system, colorectal, and gastric cancers, with no significant interactions observed. Taking the high-increasing group as an example, among participants aged <50 years, the corresponding risk of digestive system cancer (HR = 4.884, 95% CI: 1.633–14.608) was higher than that among participants aged ≥50 years (HR = 1.778, 95% CI: 1.056–7.888). Among participants with normal BMI (18.5–28 kg/m^2^), the high-increasing group was still associated with an increased risk of digestive system cancer (HR = 3.417, 95% CI: 1.643–7.146). Among participants with normal or abnormal WC, the associations between high-increasing group and the risk of digestive system cancer all showed statistical significance.

### Sensitivity analysis

Several sensitivity analyses were performed to validate the stability of the results (Table 3). Regardless of whether we excluded patients diagnosed in the first year, those taking oral hypoglycemic, lipid-lowering, or antihypertensive medications, or those with HBV infection, the associations between TyG index trajectories and digestive system cancers, colorectal cancer, and gastric cancer risk remained significant. Furthermore, both the subdistribution hazard and cause-specific risk competing risk models (Supplementary Table S7) showed that, compared with the low-stable group, participants in the moderate low-stable, moderate high-stable, high-stable, and high-increasing groups had increased risks of digestive system, colorectal, and gastric cancers.

**Table 3:** Sensitivity analysis.

Tumor types	Low-stable	Moderate low-stable	Moderate high-stable	High-stable	High-increasing
Exclude participants with cancer diagnosed within 1st year of follow-up (*n* = 52,689)^*^					
Digestive system cancers	Ref.	0.987 (0.784,1.243)	1.301 (1.012,1.672)	1.331 (1.026,1.856)	2.617 (1.526,4.488)
Colorectal cancer	Ref.	1.441 (0.920,2.255)	1.751 (1.078,2.844)	2.071 (1.109,3.866)	3.594 (1.321,9.778)
Gastric cancer	Ref.	1.100 (0.656,1.846)	1.627 (0.998,2.826)	2.253 (1.132,4.485)	3.434 (1.110,10.619)
Excluding participants taking antihypertensive drugs (*n* = 47,797)^*^					
Digestive system cancers	Ref.	1.121 (0.893,1.407)	1.439 (1.120,1.850)	1.666 (1.856,2.342)	2.567 (1.434,4.599)
Colorectal cancer	Ref.	1.479 (0.952,2.297)	1.769 (1.097,2.865)	2.388 (1.294,4.410)	3.177 (1.067,9.468)
Gastric cancer	Ref.	1.304 (0.782,2.175)	1.758 (1.007,3.067)	2.440 (1.201,4.955)	4.181 (1.347,12.977)
Excluding participants taking hypoglycemic drugs (*n* = 51,557)^*^					
Digestive system cancers	Ref.	1.111 (0.892,1.384)	1.409 (1.107,1.794)	1.498 (1.071,2.094)	2.413 (1.333,4.370)
Colorectal cancer	Ref.	1.506 (0.980,2.314)	1.799 (1.127,2.871)	2.157 (1.172,3.969)	2.437 (1.079,8.258)
Gastric cancer	Ref.	1.210 (0.743,1.970)	1.629 (0.961,2.761)	2.206 (1.123,4.336)	3.964 (1.305,12.041)
Excluding participants taking lipid-lowering drugs (*n* = 52,807)^*^					
Digestive system cancers	Ref.	1.138 (0.913,1.418)	1.430 (1.124,1.819)	1.455 (1.047,2.024)	2.248 (1.281,3.943)
Colorectal cancer	Ref.	1.581 (1.023,2.444)	1.841 (1.149,2.951)	2.035 (1.105,3.747)	3.287 (1.214,8.900)
Gastric cancer	Ref.	1.219 (0.749,1.983)	1.628 (0.998,2.839)	2.159 (1.114,4.187)	2.319 (1.063,8.107)
Further adjusted for the TyG index in 2010					
Digestive system cancers	Ref.	1.240 (0.974,1.580)	1.711 (1.252,2.337)	1.944 (1.226,3.084)	3.966 (1.926,8.169)
Colorectal cancer	Ref.	1.533 (0.962,2.445)	1.797 (0.998,3.235)	2.001 (1.012,4.638)	3.269 (0.986,12.345)
Gastric cancer	Ref.	1.504 (0.884,2.558)	2.538 (1.303,4.945)	4.187 (1.633,10.734)	8.341 (1.866,37.295)
Excluding participants with hepatitis B virus infection (*n* = 51,927)^#^					
Digestive system cancers	Ref.	1.364 (1.047,1.777)	1.742 (1.261,2.461)	1.864 (1.147,3.028)	3.793 (1.812,7.941)
Colorectal cancer	Ref.	1.582 (0.992,2.523)	1.795 (0.998,3.227)	1.925 (0.835,4.435)	3.077 (0.835,11.424)
Gastric cancer	Ref.	1.452 (0.851,2.477)	2.256 (1.155,4.404)	3.493 (1.366,8.930)	6.177 (1.393,27.404)

^*^Model was adjusted for age, sex, marital status, educational level, type of work, regular physical activity, sedentary, smoke, alcohol use, BMI, waist circumference, hypertension, diabetes mellitus, family history of cancer, CRP, salt consumption, TC, TG, gallstone disease, cirrhosis, HBV infection. ^#^Model was adjusted for age, sex, marital status, educational level, type of work, regular physical activity, sedentary, smoke, alcohol use, BMI, waist circumference, hypertension, diabetes mellitus, family history of cancer, CRP, salt consumption, TC, TG, gallstone disease, cirrhosis, HBV infection.

## Discussion

This study, conducted over a 15-year period and involving 53,350 participants from the Kailuan cohort, elucidated the relationship between TyG index trajectories and digestive system cancers. Compared with a single measurement of the TyG index, TyG index trajectories were better at predicting the risk of digestive system cancers among participants. Compared with participants in the low-stable group (TyG < 8), participants with persistently high TyG index trajectories, had a unhealthier body shape phenotype and significantly increased risk of digestive system cancers, particularly colorectal and gastric cancers.

TyG has gained widespread recognition as an indicator of insulin resistance. In some studies, the TyG index has been a better predictor of metabolic abnormalities and outcomes in adults than the traditional homeostasis model assessment of insulin resistance index (20). Several studies have explored the association of TyG with disease risk and prognosis. A meta-analysis that included six studies and 1 million participants found that, compared with participants with a low TyG index, those with a high TyG index had a 14% increased risk of cancer (21). The importance of TyG is evident in the prognosis of patients with digestive system cancers. Ruan et al. (22) showed that the TyG index was a useful indicator for predicting the prognosis of patients with colorectal cancer. In addition, a study involving 150,000 individuals demonstrated that TyG can predict the risk of cardiovascular events in cancer survivors (23). However, this relationship appears to be less robust for non-digestive system cancers. In an analysis of participants in the UK Biobank, Wang et al. (24) found that even after adjusting for multiple potential confounders, the TyG index was not significantly associated with lung cancer risk.

Unlike previous studies that relied on single measurements to assess patient prognosis, TyG, as a composite index of TG and FBG, is highly susceptible to various influencing factors that can change over time and affect prognosis (25). To our knowledge, regarding the risk of digestive system cancers, this study is the first to longitudinally record the TyG and analyze the association between its changing patterns and the risk of digestive system cancers. Notably, different TyG index trajectories may exhibit differential associations with core mechanisms underlying cancer development, which further explains the variation in cancer risk among trajectory groups. For instance, the high-increasing TyG trajectory (HR = 2.48 for digestive system cancer) and high-stable TyG trajectory (HR = 1.45) both correlate with elevated cancer risk, but their links to mechanisms like insulin resistance, lipid metabolism disorder, and chronic inflammation differ substantially. From the perspective of lipid metabolism, the high-increasing group shows a more dramatic rise in triglyceride (TG) levels (with a median TG of 7.13 mmol/L in 2010, Table 1) compared to the high-stable group (median TG of 3.38 mmol/L in 2010). This rapid TG accumulation may lead to more severe lipid toxicity, promoting the formation of ROS and oxidative damage to intestinal epithelial cells. In terms of insulin resistance, the high-increasing trajectory likely reflects progressive deterioration of insulin sensitivity: as TyG index rises continuously, hyperinsulinemia may intensify, further activating the insulin-like growth factor-1 signaling pathway to stimulate uncontrolled cell proliferation and inhibit apoptosis. In contrast, the high-stable trajectory represents a persistent but non-worsening state of insulin resistance, resulting in milder activation of oncogenic pathways and thus a lower HR.

We speculate that these results are because of the close mechanistic links between insulin resistance and digestive system cancer. As proposed in our study, changes in insulin resistance may influence the risk of digestive system cancers by altering body shape phenotypes. In this study, PC1 (symmetrical and tall body type): Individuals have large overall body dimensions; note weight-height matching. An elevated TyG index (e.g., high-stable trajectory) may raise metabolic load via simultaneous muscle/fat increase, requiring body composition testing for cancer risk assessment. PC2 (hip circumference-dominant type): Significantly lower waist-hip ratio (WHR), indicating higher hip fat (especially subcutaneous fat); highest proportion (42.0%) in low-stable TyG trajectory with low metabolic risk. PC3 (stocky central obese type): Combines high BMI and central obesity, accounting for 34.1% of high-stable TyG individuals (vs. 6.9% in low-stable group); monitor insulin resistance (e.g., TyG index, fasting insulin) as it correlates with significantly higher digestive system cancer risk. PC4 (occult central obese type): Normal BMI but abnormal circumference indicators (easily missed by traditional BMI), identified via WHR; abnormal abdominal/hip fat distribution may increase cancer risk by exacerbating insulin resistance (elevated TyG index). This aligns with previous research, where a study using machine learning methods confirmed the role of WC in the occurrence of digestive system cancers (26). Additionally, research has demonstrated a close association between body shape phenotypes of central obesity and an increased risk of cancer-specific mortality (27).

This relationship also involves the interactions between multiple biological processes and molecular mechanisms. First, insulin resistance is often accompanied by high insulin levels. Hyperinsulinemia and associated diabetes are risk factors for digestive system cancers, particularly colorectal, biliary tract, and pancreatic cancer (28–30). In contrast, insulin resistance is closely associated with an imbalance in the insulin-like growth factor (IGF) axis (31). When insulin resistance and hyperinsulinemia are present, IGF receptors are overly activated, leading to a cascade of downstream PI3K/AKT and RAS/MAPK signaling, causing uncontrolled cell proliferation (32). Additionally, excessive activation of IGF receptors can alter the p53 pathway to suppress apoptosis-related signaling, thereby increasing cell survival (33). Second, insulin resistance is often associated with abnormalities in lipid metabolism. Abnormalities in lipid metabolism are accompanied by the accumulation of chronic inflammation, which is a key feature of cancer. Inflammation affects cancer development through various mechanisms, including promotion of cell proliferation, inhibition of apoptosis, induction of DNA damage and mutations, and immune evasion (34). In contrast, abnormalities in lipid metabolism can lead to increased lipid breakdown and elevated oxidative stress levels, resulting in the production of reactive oxygen species and oxidative lipids, which can damage DNA and affect cell growth and differentiation (35, 36). Finally, insulin resistance often coexists with glucose metabolism disorders, which are characteristic features of cancer. Higher glucose uptake rates, increased glycolysis, and reciprocal promotion of insulin resistance contribute to the accelerated development of digestive system cancers (37).

However, this study has several limitations. First, although it was a prospective observational study, the results do not provide sufficient evidence to establish a causal relationship between TyG and the incidence of digestive system cancers. Future research should explore and validate this causal relationship using rigorous, multicenter, prospective cohort studies. Second, this study is based on the Kailuan cohort, which consists mainly of male workers from the coal mining and manufacturing industries. The sex imbalance may limit the generalizability of the results (38). Third, although we adjusted the models for BMI and waist circumference, we did not collect detailed information on the participants’ body composition or body shape. Some studies have reported that differences and trajectory in body shape can also influence metabolic patterns and cancer risk (39, 40). Fourth, for cancers such as pancreatic cancer and esophageal cancer, due to their low incidence, the study may have failed to yield significant results. We are not certain whether this is caused by insufficient sample size or differences in biological mechanisms, and future studies with larger sample sizes are needed to supplement the evidence. Finally, due to data limitations, we did not include dietary details (such as dietary fiber and red meat intake), gut microbiota, and other factors known to affect digestive system cancers, which may lead to bias (41, 42).

## Conclusion

In summary, compared to a single measurement of the TyG index, the TyG index trajectory serves as a better predictive indicator of the risk of digestive system cancers. The TyG index trajectory is closely associated with the risk of digestive system cancers, possibly due to the correlation between higher levels of TyG index and less healthy body shape phenotypes. Longitudinal monitoring of changes in the TyG index effective control of lipid and blood glucose levels are crucial for preventing digestive system cancers.

## Data Availability

The original contributions presented in the study are included in the article/Supplementary material, further inquiries can be directed to the corresponding authors.

## Footnotes

^1^ https://bodyvisualizer.com/
